# Alizarin, which reduces ExoS, attenuates inflammation by *P. aeruginosa* in H292 cells

**DOI:** 10.71150/jm.2411012

**Published:** 2025-05-27

**Authors:** Seung-Ho Kim, Hye In Ahn, Jae-Hoon Oh, Da Yun Seo, Jung-Hee Kim, Ok-kyoung Kwon, Ji-Won Park, Kyung-Seop Ahn

**Affiliations:** 1 Natural Medicine Research Center, Korea Research Institute of Bioscience and Biotechnology, Chungbuk 28116, Republic of Korea; 2 College of Life Sciences and Biotechnology, Korea University, Seoul, 02841, Republic of Korea; 3 ELEO Inc., Daejeon 34141, Republic of Korea; 4 Stem Cell Convergence Research Center, Korea Research Institute of Bioscience and Biotechnology, Daejeon 34141, Republic of Korea; 5 AptaBio, Gyeonggi-do 16954, Republic of Korea; 6 Division of Practical Research, Honam National Institute of Biologucal Resources (HNIBR), Jeollanam-do 58762, Republic of Korea; 7 Advanced Research Center for Island Wildlife Biomaterials, Honal Natinal Institute of Biological Resources, Jeollanam-do 58762, Republic of Korea

**Keywords:** *Pseudomonas aeruginosa*, Type 3 secretion system (T3SS), H292, NF-κB, acute infection, alizarin

## Abstract

*Pseudomonas aeruginosa* (*P. aeruginosa*) is resistant to several drugs as well as antibiotics and is thus classified as multidrug resistant and extensively drug resistant. These bacteria have a secretion system called the "type 3 secretion system (T3SS)", which facilitates infection by delivering an effector protein. ExoenzymeS (ExoS) is known to induce cell death and activate caspase-1. In particular, patients infected with *P. aeruginosa* develop diseases associated with high mortality, such as pneumonia, because no drug targets an ExoS or T3SS. We selected natural compounds to treat T3SS-mediated pneumonia and chose alizarin, a red dye. We confirmed the effects of alizarin on T3SS by bacterial PCR and ELISA. It was confirmed that alizarin regulates ExoS by inhibiting *exsA* but also *popD* and *pscF*. Furthermore, in infected H292 cells, it not only attenuates inflammation by inhibiting lipopolysaccharide (LPS)-induced phosphorylation of nuclear factor kappa-light-chain-enhancer of activated B cells (NF-κB) p65 but also interferes with the level of ExoS delivered into the host and modulates caspase-1. We confirmed this result and determined that it led to decreases in proinflammatory cytokines such as Interleukin-1beta (IL-1β), Interleukin-6 (IL-6), and Interleukin-18 (IL-18). Therefore, we suggest that alizarin is a suitable drug for treating pneumonia caused by *P. aeruginosa* because it helps to attenuate inflammation by regulating T3SS and NF-κB signaling.

## Introduction

The quality of human life has improved over time due to many factors, one of which is the development of antibiotics. There is no doubt that antibiotics affect bacteria, but the abuse of antibiotics has led to the emergence of several multidrug-resistant bacteria that overcome antibiotics over time. Among them, *Pseudomonas aeruginosa* (*P. aeruginosa*) is resistant to several drugs as well as antibiotics, so it is classified as multidrug resistant (MDRPA) and extensively drug resistant (XDRPA) (Kang et al., 2021; Luna et al., 2013; Tummler, 2019). In the case of lung diseases such as pneumonia caused by *P. aeruginosa*, there is currently no suitable treatment, it requires a high cost and long treatment time, and a cure is difficult (Morales et al., 2012). Most patients with weakened immunity become infected with *P. aeruginosa* as a secondary infection in the hospital (Sadikot et al., 2005).

The reason for this is the type 3 secretion system (T3SS), a weapon that contains a needle-like complex for subjugating the host to *P. aeruginosa* infection. It is regulated by the "ExsA" of the AraC family, transcription factors that are regulated in host contact or environments such as low Ca^+^ and deliver exotoxins directly to the host (Diaz et al., 2011; Marsden et al., 2016; Shrestha et al., 2015). The currently characterized exotoxins known (ExoS, ExoT, ExoU, ExoY) include four types that are known to cause rapid death of phagocytes and a hyperinflammatory response in the host (Azimi et al., 2016; Kaminski et al., 2018; Zhang et al., 2019). In lung infection caused by *P. aeruginosa*, the host cells fail to perform a normal immune response due to ExoS attacking cells, resulting in nuclear factor κB (NF-κB) phosphorylation (Cohen and Prince, 2013; Ulland et al., 2015). As a result, the levels of inflammatory cytokines such as interleukin-1β (IL-1β) and interleukin-18 (IL-18) are overexpressed in the host cells. The produced cytokines are converted to their mature forms by inflammasome/NLR family CARD domain containing 4 protein (NLRC4), and NLR family pyrin domain containing 3 (NLRP3) is activated as exotoxin and released into the extracellular environment. Mature IL-1β accumulation indicates lung damage that results from neutrophils recruited by IL-1β. Additionally, mature IL-18 acts on Natural killer (NK) cells and T cells to induce interferon (IFN)-γ synthesis and on monocytes to induce Granulocyte-Macrophage Colony-Stimulating Factor human (GM-CSF), tumor necrosis factor (TNF)-α, and IL-1β expression. Thus, a continuous increase in inflammatory cytokines induces a continuous inflammatory response in the host (Cohen and Prince, 2013; Kaminski et al., 2018; Ta and Vanaja, 2021). The immune response is not normal during the inflammatory response and can result in the development of a serious lung infection.

We selected alizarin as a candidate for the prevention of lung infections by *P. aeruginosa*. Alizarin is abundantly present in the roots of the genus Rubia, which are readily found all over the world except in cold climates. In China, since ancient times, rubia root has been used to treat hemostasis, menstruation, fractures, and mycotoxins. We hypothesized that alizarin was responsible for this effect. Recently, various applications of alizarin have been reported in medicine and industry, but no research has evaluated its effects on *P. aeruginosa* infection (Trovato et al., 2022; Xu et al., 2022; Zhang et al., 2021).

We found that the alizarin inhibits infection through the T3SS of *P. aeruginosa* by inhibiting the NF-κB pathway through a cytokine related to inflammation. Therefore, this study suggests that alizarin can prevent the progression from infection by inhibiting the expression of T3SS both in vitro and at the bacterial level.

## Materials and Methods

### Source of alizarin

We purchased alizarin, which has a molecular weight of 240.21 and a molecular formula of C_14_H_8_0_4_ (Cat. No. 72-48-0, FUJIFILM Wako Pure Chemical Corporation, USA), from Wako Chemical.

### Plasmid and strain information

All strains and plasmids were provided by Korea University and Natural Medicine Research Center. All materials were derived to use in this study from *P. aeruginosa* (PA) are listed in Table 1. Some plasmids were transformed into the *P. aeruginosa* *K* wild-type strain (PAK) to create mutant strains. PAK exoST::Ω/pHW0225 (p137) is a mutant strain that produces and secretes ExoS-Flag and was used to measure ExoS. The PAK *exsA*::Ω/pHW0029 (p34) strain is a negative strain constructed to exhibit defects in the entire T3SS. Additionally, PAK-ΔSTmt-pUCP18 (PAK ExoS) is a mutant strain constructed to secrete only ExoS.

### Bacterial culture

PAK was provided by the University of Florida, and the mutated strain was provided by Dr. Ha from Korea University. First, we used an luria-bertani broth (LB) plate containing 1.5% tryptone (BD Bacto^TM^ Tryptone, Cat. No. 211705, BD, USA), 1.5% NaCl (sodium chloride, Cat. No. 19015S0350, JUNSEI, Japan), yeast extract (Bacto^TM^ Yeast Extract, Cat. No. 212750, BD, USA), and agar (Bacto^TM^ Agar, Cat. No. 214010, BD, USA). Before autoclaving for sterilization, components were mixed with sterilized distilled water. The LB agar was slowly cooled at room temperature (RT), and the antibiotics were added once it had reached a suitable temperature. The LB agar was poured into plates for bacterial culture and was then hardened. The strain was inoculated on an LB plate into 3-way streaking for one day at 37℃ in a shaker incubator. Next, a single colony was obtained and inoculated into a 14 ml round tube (Cat. No. 40014, SPL, Korea) with LB broth and antibiotics (Table 1). Each strain required LB broth but different antibiotics. After incubating at 37℃ in a shaker incubator at 200 RPM for 16 h, new 14 ml round tubes were inoculated with fresh LB and alizarin (2.5, 5, 10, and 20 μM). Antibiotics and 5 mM ethylene glycol bis(2-aminoethyl ether)-N,N,N',N'-tetraacetic acid (EGTA, C_14_H_24_N_2_O_10_) were added, and bacteria were incubated and diluted 1:1,000. The bacteria were then incubated under the same conditions for use in the bacterial experiments.

### Biofilm formation suppression

The biofilm formation measurement method was performed using crystal violet. A single PAK colony was collected and cultured in a 14 ml round tube with LB and antibiotics for 16 h in a 37℃ shaker incubator at 200 RPM. Incubated bacteria were diluted in fresh LB at a 1:1000 ratio and then incubated again under the same conditions.

Bacteria were cultured in 96-well plates with LB and alizarin (2.5, 5, 10, and 20 μM), while the positive control was treated with ciprofloxacin in place of alizarin, and the negative control was treated with only bacteria. Each well volume was set to 200 μl. The concentration of bacteria was 0.1 OD, and the concentration of ciprofloxacin was 0.02 mg/ml. After the 96-well plate was incubated in a 37℃ incubator for 16 h, it was washed with distilled water for a total of 3 cycles. Then, the 96-well plate was stained with 1% crystal violet (CV; Crystal violet, Cat. No. C3886-25G, Sigma, USA) and reacted for 15 min at RT, after which the wash step was repeated. The 96-well plate was dried at 60℃ in an oven for 1 h, and then 70% ethanol was added to each well to allow the biofilm to dissolve. For biofilms that were attached to the well walls, the wells were stirred until the biofilms had dissolved in ethanol. To detect biofilms, OD measurements were performed at an OD of 585 nm using a multifunctional microplate reader (SPARK 10M, Tecan, Switzerland).

### ExoS ELISA

ExoS ELISA was designed to detect ExoS expression levels in bacteria by Dr. Ahn (Kim et al., 2005). In this study, we used a p137 strain that expressed only ExoS-FLAG (Table 1). First, a customized anti-ExoS antibody from Koma Biotech was diluted 1:5,000 in carbonate-bicarbonate buffer (Carbonate-Bicarbonate Buffer, Cat. No. C3041-50CAP, Sigma, USA), and the coating step proceeded at 4℃ overnight in 96-well microplates. After 3 cycles of washing with wash buffer, the plate was blocked with 2% bovine serum albumin (BSA) (Cat. No. 30063-572, Gibco, New Zealand) in PBS for 1 h at RT. The wash step was repeated, and the bacterial supernatant was added to the well. The plate was incubated at RT for 2 h and washed for a total of 5 cycles. Anti-mouse anti-FLAG antibody (anti-DDDDK antibody; Cat. No. ARG62342, Arigo Biolaboratories, Taiwan) was diluted to 1:5,000 in 1% bovine serum albumin. Horseradish peroxidase (HRP)-conjugated goat anti-mouse immunoglobulin G (IgG) (Cat. No. 115-035-003, Jackson, USA) diluted to 1:2,000 in 1% BSA was added to the well for 1 h. After the reaction was complete, the wash step was repeated, and a substrate solution was added. When the color changed from white to yellow, the reactions in the plates were stopped with H_2_SO_4_, and the absorbance was measured at 450 nm using a multifunctional microplate reader (SPARK 10M, TECAN, Switzerland) including reference data at 570 nm.

### Cell culture

H292 cells (human epithelial cells derived from human lung carcinoma) were purchased from American Type Culture Collection (ATCC, USA). H292 cells were cultured in RPMI 1640 medium (Cat. No. LM 011-01, WELGENE, Korea) supplemented with 10% fetal bovine serum (FBS; Invitrogen, USA) and antibiotics (1x penicillin‒streptomycin; Invitrogen, USA) and incubated at 37℃ in a 5% CO_2_ incubator.

### Cell viability

Cell viability was assessed in an MTT assay (3-(4,5-dimethylthiazol-2-yl)-2,5-diphenyl tetrazolium bromide assay; Amresco, USA). First, H292 cells were seeded at a concentration of 1 × 10^5^ cells/ml. After incubation for 4 h at 37℃ in a 5% CO_2_ incubator, H292 cells were treated with alizarin (2.5, 5, 10, and 20 μM) and ampicillin (5 μg/ml; Cat. No. AMP25, LPS solution, Korea) diluted in PBS. The control group was treated with DMSO (SAMCHUN, Cat.000D2855). The alizarin-treated plate was incubated in a 37℃ 5% CO_2_ incubator for 20 h. After 20 h of reaction, 5 μl of 5 mg/ml MTT solution was added to each well, and the plate was incubated at 37℃ for 4 h. After the remaining medium was removed, 100 μl DMSO was added to each well. For optical density measurements, the OD was read at 570 nm using a multifunctional microplate reader (SPARK 10M, Tecan, Switzerland).

### Cytokine ELISA

The cytokine expression levels were measured by ELISA in H292 cells. First, H292 cells were seeded into 12-well plates and incubated for 24 h. Next, the medium was changed to RPMI 1640, including free FBS, after washing each well with PBS. After again incubating the plate for 1 h, each well was treated with alizarin, and the plate was incubated at 37℃ for 1 h. PAK ExoS single colonies were incubated in a 14 ml round tube containing diluted carbenicillin (Cat. No. 2485-10G, BioVision, USA) and LB broth. After one day, the incubated ExoS strain was diluted in a 14 ml round tube to 1:1,000. Infection was performed at an MOI of 100. OD 600 nm measurements were taken after 16 h of incubation in a 37℃ incubator. H292 was incubated at 37℃ for 1 h 30 min, and every well was washed with DPBS containing diluted tobramycin (Cat. No. T-4014, Sigma, USA) in a 37℃ incubator for 15 min twice.

Finally, RPMI 1640 without FBS was added to each well, and the cells were allowed to react for approximately 4 h. The H292 supernatant was collected. For cytokine measurement, the supernatant was used in an ELISA kit following the steps of an ELISA manual, and the cytokines measured were IL-1β (Cat. No. 557953, BD, USA), IL-18 (Cat. No. DY318-05, R&D, USA), IL-8 (Cat. No. DY208, R&D, USA), and IL-6 (Cat. No. 55520, BD, USA).

### Real-time PCR analysis for observation of mRNA expression at the bacterial and cell levels

Bacterial RNA isolation was performed for 2 days. First, PAK ExoS cells were inoculated in a 14 ml round tube with LB broth and antibiotics, but the ST deletion strain was not treated with antibiotics. Inoculated bacteria were incubated for 16 h at 37℃ in a 200 RPM shaking incubator. After the incubation, new 14 ml round tubes were supplemented with alizarin at concentrations of 2.5, 5, 10, and 20 μM. Additionally, fresh LB broth was added together with antibiotics, and the cultures were diluted at a 1:1000 ratio and then incubated again under the same conditions.

For cell RNA isolation, H292 cells were incubated in a 12-well plate for 24 h at 5 × 10^5^ cells/ml at 37℃ in a 5% CO_2_ incubator. H292 cells were changed to fresh RPMI 1640 medium, and alizarin was added. Next, H292 cells were infected with pucp18 diluted in DPBS for 1 h 30 min. To eliminate the remaining bacteria, every well was washed using DPBS supplemented with tobramycin.

Both bacterial and cell RNA isolations were performed through the chloroform/phenol RNA extraction method using TRIzol. Extracted RNA was synthesized into complementary DNA using a TOYOBO cDNA kit; thus, real-time PCR (polymerase chain reaction) was performed with primers and SYBR Green, and the primers used are shown in Table 2.

### Whole-protein analysis of infected cells by Western blot

H292 cells infected with PAK ExoS were seeded in a 12-well plate at 5 × 10^5^ cells/ml and cultured. After culturing for one day, the existing medium was removed and replaced with pure RPMI 1640 (free FBS), followed by a reaction for 1 h. Alizarin (2.5, 5, 10, and 20 μM) was added and reacted again under the same conditions. Cells were infected for 1 h using PAK ExoS at an MOI of 100. After removing the remaining medium, 40 μl of whole-cell lysis buffer (Protein extraction solution; NP-40, Cat. No. EBA-1049, ELPIS-Biotech, Korea) including protease inhibitor and phosphatase inhibitor was added to each well and reacted for 3 min on a shaking rocker. Whole-cell debris was collected in an Eppendorf tube and centrifuged at 13,000 RPM for 3 min at 4°C.

The extracted proteins were loaded on a 10% sodium dodecyl polyacrylamide gel and separated by electrophoresis. After electrophoresis, the proteins were transferred using a hydrophilic polyvinylidene fluoride membrane (Cat. No. IPVH00010, EMD Millipore, USA). The membrane was blocked using 5% skim milk, and p65 (Cat. No. 8242S, Cell Signaling, USA), p-p65 (Cat. No. 3033S, Cell Signaling, USA), IKKβ (Cat. No. 8943s, Cell Signaling, USA), p-IKKα/β (Cat. No. 2697S, Cell Signaling, USA), NMRP3 (Cat. No. 15182s, Cell Signaling, USA), NMRC4 (Cat. No. 12421S, Cell Signaling Technology, USA), Caspase-1 (Cat. No. 3866S, Cell Signaling, USA), and β-actin (Cat. No. SC-4778, Santa, USA) antibodies were reacted at 4°C for one day. The membrane was then washed using TBS-T buffer, and the secondary antibody was reacted at room temperature for approximately one hour. For protein detection, an ECL solution kit (PierceTM ECL Western Blotting Substrate, Cat. No. 32106, Thermo Fisher, USA) was used, and LAS-4000 (Fujifilm, Japan) was used for imaging.

### Protein analysis of p65 translocation via western blot analysis of the nuclear fraction

We confirmed the p65 level when NF-κB was activated by measuring p65 in the nucleus. Nuclear extraction was performed using a nucleus extraction kit (Cat. No. 2900, EMD Millipore, USA). After making a cell pellet, foregoing the western blot step, cytosolic protein was obtained using a cytosol extract solution and washed with 1 × PBS. A pellet was again generated through centrifugation, and nuclear protein was extracted using a nucleus extract solution. The electrophoresis step was the same as the western blot for whole protein and for detecting p65 antibody using rabbit antibody (horseradish peroxidase (HRP)-conjugated goat anti-rabbit immunoglobulin G (IgG), Cat. No. 111-035-003, Jackson, USA).

### Protein analysis of ExoS-Flag through western blot in *P. aeruginosa*

To measure ExoS-Flag in bacteria through western blotting, we used a p137 strain and p34. Each strain was incubated for one day and supplied fresh LB with alizarin (2.5, 5, 10, and 20 μM), antibiotics, and 5 mM EGTA to induce ExoS-Flag. After generating a pellet through centrifugation, we melted the pellet with NP40. To obtain protein, we performed centrifugation (14,000 RPM, 10 min, 4°C) and BCA quantification. The Western blot step was the same as that used for other protein analyses, but the antibody used was diluted 1:1000 with 0.1 mg/ml anti-ExoS Flag. 

## Results

### Effect of alizarin on *Pseudomonas aeruginosa*

**Fig. 1.**
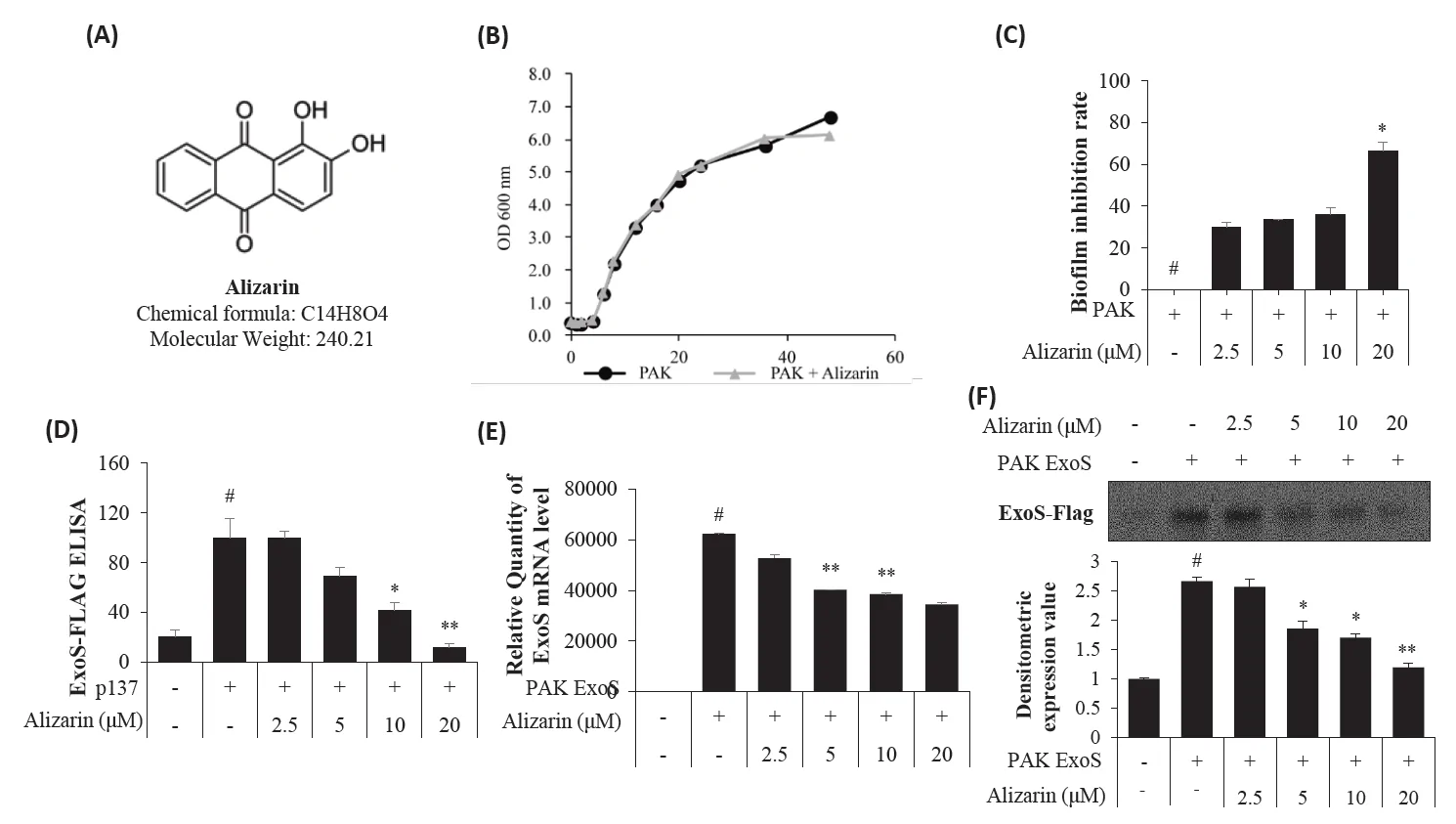
Effect of alizarin on *P. aeruginosa*. (A) Compound structure of alizarin. (B) Bacterial growth curve with and without 20 μM alizarin. (C) Biofilm activity evaluated using crystal violet (CV) and treated with alizarin (2.5, 5, 10, and 20 μM). The control group was treated with 0.02 mg/ml ciprofloxacin (CIP). (D) ExoS-Flag ELISA was designed to detect ExoS in p137 and p34. ELISA was used in a supernatant incubated with p137 and p34, including LB broth and 5 mM EGTA. (E) RNA extraction was performed by culturing PAK ExoS with LB broth, 5 mM EGTA and alizarin (2.5, 5, 10, and 20 μM). (F) To detect ExoS in H292 cells infected with the p137 strain, Western blotting was performed after incubation with the PAK ExoS strain, and a 10% polyacrylamide gel was used. The antibody used was diluted 1:1,000 with anti-ExoS-Flag in 5% skim milk. The margin of error is presented as the mean ± SD of at least two experiments. The positive control is represented by "#" when compared to the normal group when P < 0.01. Significant differences from the positive control: ^*^ P < 0.05, ^**^ P < 0.01, ^***^ P < 0.001. PAK exoST::Ω/pHW0225 (p137; double mutated ExoS-FLAG), PAK *exsA*:: Ω/pHW0029 (p34; PAK with the malfunction of T3SS transcription control gene loci with Ω cassette), PAK-ΔSTmt-pUCP18 (PAK ExoS; PAK-ΔSTmt recovered ExoS gene).

To investigate the effect of alizarin on *P. aeruginosa*, several factors were identified by culturing bacteria with alizarin. Depending on the presence or absence of alizarin, the p137 strain was cultured for 20 h to confirm the bacterial growth curve, and there was no difference between the two groups (Fig. 1B). Next, it was confirmed that the biofilm, which is known to contribute to host infection, was dose dependently inhibited according to the alizarin concentration when observed in the presence or absence of alizarin in PAK (wild-type) (Fig. 1C). ELISA was performed using ExoS-FLAG to observe the change in ExoS delivered to the host through the T3SS. Alizarin showed a high inhibitory effect at a high concentration of 20 μM (Fig. 1D). Additionally, real-time PCR and western blotting were performed to determine where the inhibitory effect originated (Fig. 1E and 1F). The results confirmed that alizarin reduced production by inhibiting ExoS at the mRNA level. Through this result, we expected that the ExoS inhibitory effect of alizarin might have been shown by affecting other T3SS genes.

### Alizarin reduces the levels of other T3SSs by regulating the mRNA level of ExsA

**Fig. 2.**
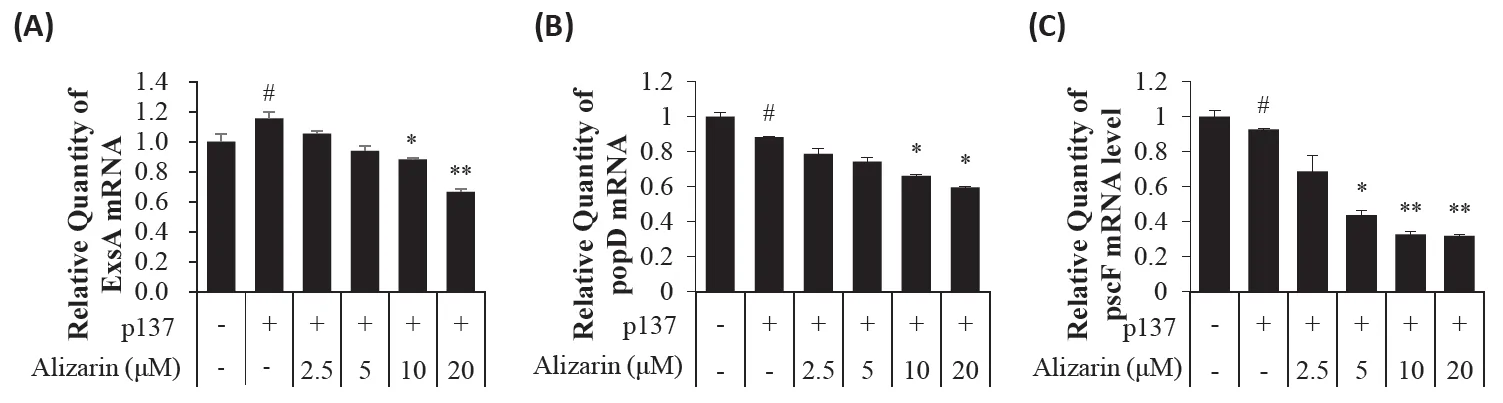
Alizarin inhibits the T3SS mRNAs *ExsA*, *pscF*, and *popD* in *P. aeruginosa*. Real-time PCR was performed by incubating the PAK ExoS and ΔST strains. Each strain was incubated at 37℃ in a shaker incubator for 24 h and supplied with fresh LB broth, 5 mM EGTA, and alizarin (2.5, 5, 10, and 20 μM). The extracted RNA of each group was equally quantified. (A) The mRNA expression of *ExsA*, which regulates T3SS, was decreased when alizarin was added. (B) The mRNA expression of *popD*, which forms the pore of the host's cell membrane, was decreased when 10 and 20 μM alizarin was added. (C) The mRNA expression of *pscF*, which plays a conduit role, was decreased when 5, 10 and 20 μM alizarin was added. The margin of error is presented as the mean ± SD of at least two experiments. The positive control is represented by "#" when compared to the normal group when P < 0.01. Significant differences from the positive control: ^*^ P < 0.05, ^**^ P < 0.01, ^***^ P < 0.001. PAK-ΔSTmt-pUCP18 (PAK ExoS; PAK-ΔSTmt recovered the ExoS gene), PAK-ΔSTmt (ΔST; double effector mutant: functional needle and translocon apparatus without a known effector).

We noted the decreased secretion and production levels of ExoS in previous experiments (Fig. 1D and 1E). Therefore, to determine where the decrease in ExoS originated, the mRNA levels of the components of the T3SS were confirmed through real-time PCR. The first element, ExsA, is a transcriptional regulator known to upregulate the T3SS gene. The *ExsA* levels of bacteria decreased according to the concentration of alizarin (Fig. 2A).

Next, we investigated a factor known to form pores in the host cell membrane in T3SS, termed *popD*. Alizarin dose dependently decreased the mRNA level of *popD* at high concentrations of 10 μM and 20 μM (Fig. 2B). Next, *pscF* was investigated as a factor that acts as a conduit for the delivery of effector proteins such as ExoS into the host. It was confirmed that alizarin showing a decreasing pattern the mRNA level of *pscF* (Fig. 2C). These results could be expected to follow because *ExsA*, which is involved in the overall regulation of T3SS, decreased, suggesting that ExoS is also involved.

### Alizarin reduces the protein level of ExoS in infected H292 cells

**Fig. 3.**
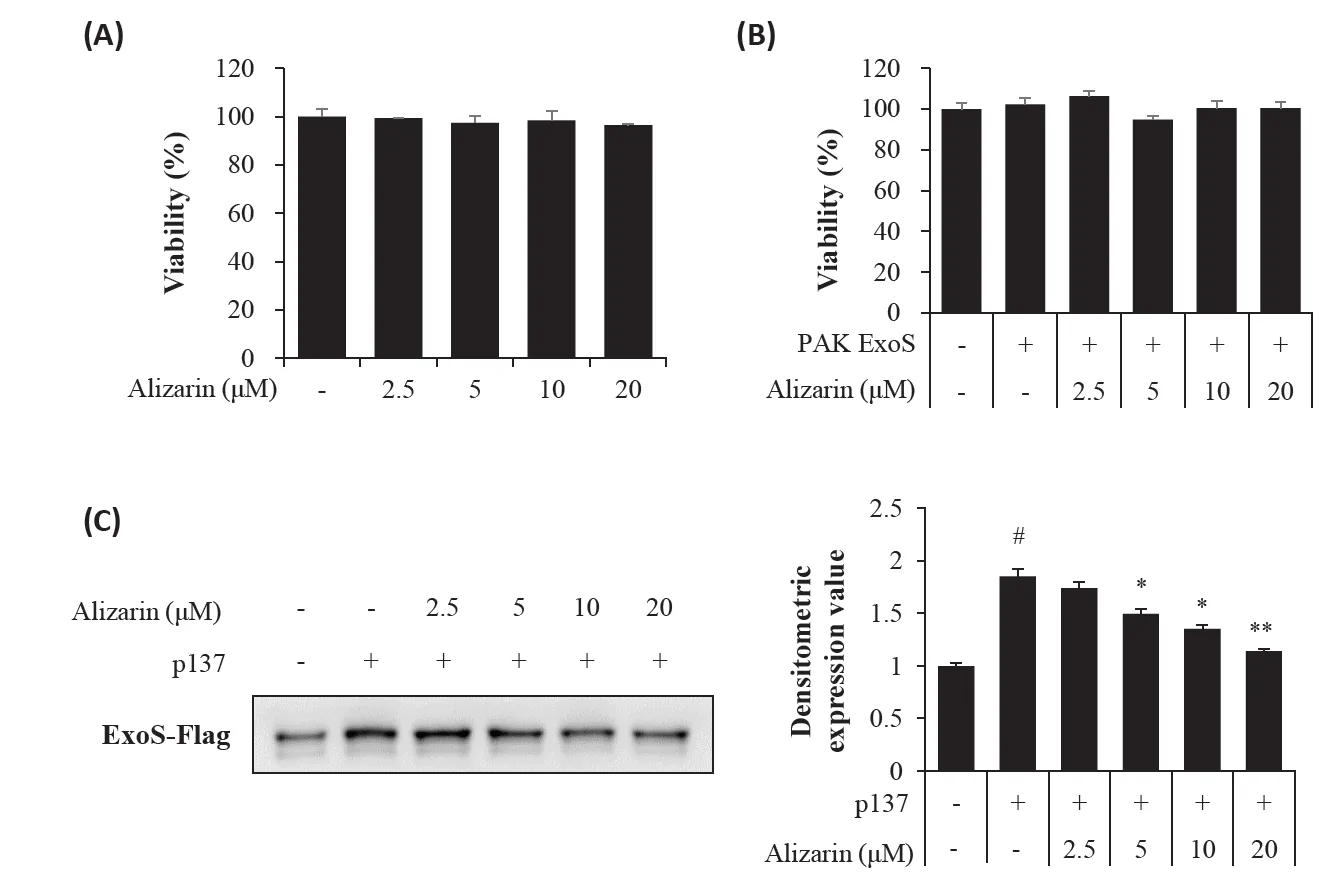
Effect of alizarin on H292 cells and the reducing effect of ExoS. (A) H292 cells were cultured at 1 × 10^5^ cells/ml in 96-well plates, and an MTT assay was performed to measure cytotoxicity to alizarin. The addition of alizarin (2.5, 5, 10, and 20 μM) had no effect on H292 cells. (B) H292 cells were cultured at 1 × 10^5^ cells/ml in 96-well plates and infected with *P. aeruginosa* (MOI 100) for 1 h 30 min. Cytotoxicity was not observed through the MTT assay with or without alizarin (2.5, 5, 10, and 20 μM). (C) H292 cells were cultured at 5 × 10^5^ cells/ml in 12-well plates and incubated for 24 h. Alizarin (2.5, 5, 10, and 20 μM) was added after starvation for 1 h before infection with p137 (MOI 100). ExoS-Flag was detected using an anti-ExoS-Flag antibody. PAK-ΔSTmt-pUCP18 (PAK ExoS; PAK-ΔSTmt recovered the ExoS gene). The margin of error is presented as the mean ± SD of at least two experiments. The positive control is represented by "#" when compared to the normal group when P < 0.01. Significant differences from the positive control: ^*^ P < 0.05, ^**^ P < 0.01, ^***^ P < 0.001.

We wanted to determine whether the reduced levels of ExoS induced by alizarin at the bacterial level were also expressed in cells. We first measured the cytotoxicity to determine the appropriate concentration of alizarin in H292 cells (Fig. 3A). When cells were treated with the concentration (2.5, 5, 10, and 20 μM) previously used at the bacterial level, no lethal cell concentration was observed. Additionally, it was confirmed that in H292 cells infected with PAK ExoS, treatment with alizarin at the same concentration as before did not have any effect (Fig. 3B). After that, we added alizarin to measure ExoS in cells, and after infection using the p137 strain, we measured the protein level of ExoS-Flag (Fig. 3C). The results confirmed that the level of ExoS-Flag in the cell was decreased according to the concentration of alizarin. This suggests that T3SS may also result in inhibition by alizarin at the cellular level, as observed at the bacterial level.

### Alizarin can inhibit both the production and secretion of cytokines

**Fig. 4.**
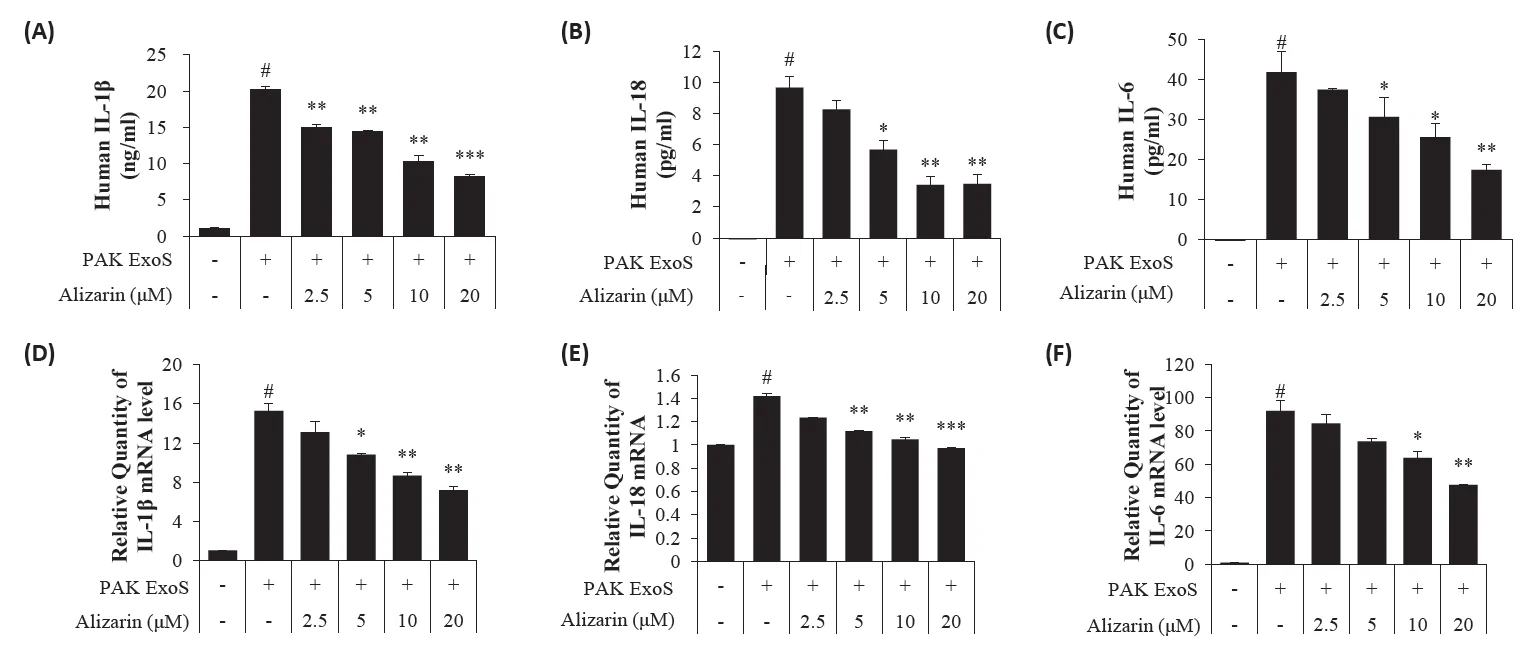
Inhibition of proinflammatory cytokines by alizarin at the mRNA and protein expression levels. Proinflammatory cytokines were measured by ELISA and real-time PCR. Supernatant and mRNA were collected from H292 cells that were treated with alizarin (2.5, 5, 10, and 20 μM) before infection with PAK ExoS (MOI 100). (A–C) The expression levels of proinflammatory cytokines were decreased when alizarin was added. (D–F) The mRNA levels of proinflammatory cytokines indicated the same result. The margin of error is presented as the mean ± SD of at least two experiments. The positive control is represented by "#" when compared to the normal group when P < 0.01. Significant differences from the positive control: ^*^ P < 0.05, ^**^ P < 0.01, ^***^ P < 0.001. PAK-ΔSTmt-pUCP18 (PAK ExoS; PAK-ΔSTmt recovered the ExoS gene).

Currently, T3SS-induced pneumonia is a well-known infection. Excessive recruitment of neutrophils due to LPS initiates NF-κB, and the formation of ExoS-activated inflammasomes proceeds. Therefore, we investigated whether alizarin could attenuate the inflammatory response by measuring the proinflammatory cytokines produced and secreted during this process (Fig. 4). We performed ELISA to measure secreted cytokines, and IL-1β was decreased in a dose-dependent manner when alizarin was added (Fig. 4A). Additionally, IL-18 indicated the same result but had no effect on H292 cells treated with 2.5 μM alizarin (Fig. 4B). Thus, alizarin was confirmed to inhibit the secretion of cytokines that induced inflammation in H292 cells. Additionally, we confirmed whether alizarin influenced the mRNA levels of cytokines through real-time PCR. The mRNA level of IL-1β showed a tendency to decrease according to the concentration of alizarin, and it was confirmed that IL-18 and IL-6 showed the same results (Fig. 4D, 4E, and 4F). These findings suggest that alizarin may affect the signaling pathway that activates proinflammatory cytokines in H292 cells infected by PAK ExoS.

### Alizarin inhibits the translocation of NF-κB and modulates the activity of Caspase-1

**Fig. 5.**
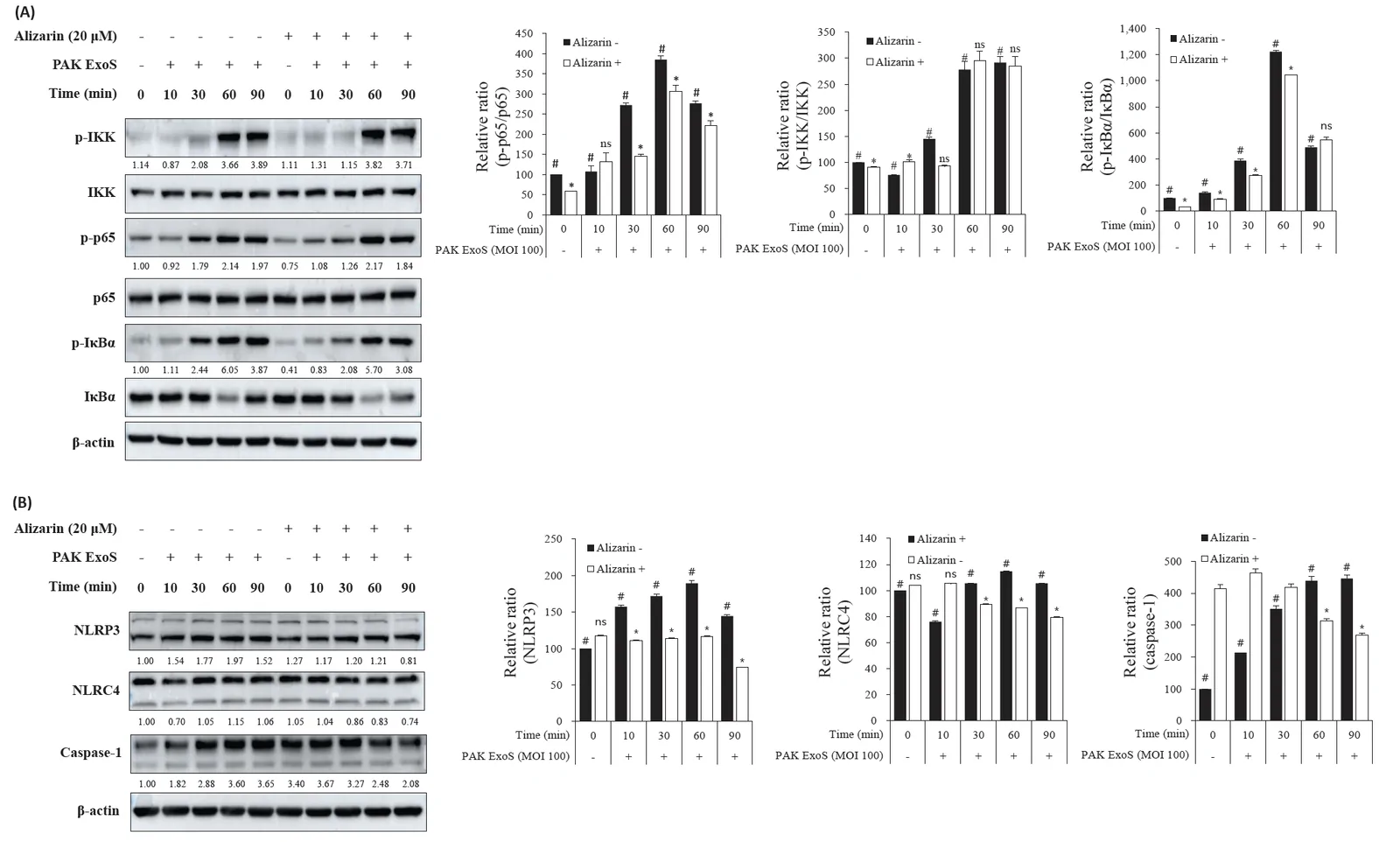
Expression of NF-κB and inflammasome by alizarin according to time. Western blotting was performed to observe NF-κB or inflammasomes (NLRP3, NLRC4, or Caspase-1). Each band differs by the infection time and the presence or absence of alizarin, but the treated concentration is the same in the case of added alizarin. (A) p65 and caspase-1, which are NF-κB factors and activators of proinflammatory cytokines, were affected by alizarin. (B) Western blotting was performed, which confirmed the levels of p65 translocation in the cytoplasm and nucleus when mediated by *P. aeruginosa* infection in H292 cells. Each band was quantified using ImageJ and is represented by a number indicating the difference between the control and nontreated bands. PAK-ΔSTmt-pUCP18 (PAK ExoS; PAK-ΔSTmt recovered the ExoS gene).

We investigated the NF-κB signaling pathway, which is activated by *P. aeruginosa* and is known to produce proinflammatory cytokines. Changes in NF-κB factors over time with and without alizarin treatment were confirmed through western blot. The results confirmed that alizarin inhibited LPS-induced p65 phosphorylation by approximately 30% when alizarin was added 1 h after infection (Fig. 5A). This effect is thought to be mediated by inhibiting the phosphorylation of IκBα, which is known to inhibit p65, and the effects on both factors were most dramatic when alizarin was added for 30 min (Fig. 5A). Additionally, in the case of caspase-1, which is known to be activated by ExoS, it was confirmed that when cells were treated with alizarin for 60 and 90 min, the protein levels in the infected cells were reduced to 30% and 43%, respectively (Fig. 5A). In addition, to confirm the decreased phosphorylation of p65, the level of p65 translocated into the nucleus was measured, and it was confirmed that the effect was evident when alizarin was added for 30 min (Fig. 5B). From the overall results, it can be expected that alizarin reduces cytokine production and secretion by inhibiting caspase-1 activated by ExoS while regulating the phosphorylation of the p65 factor.

### Alizarin inhibits the conversion of IL-1β

**Fig. 6.**
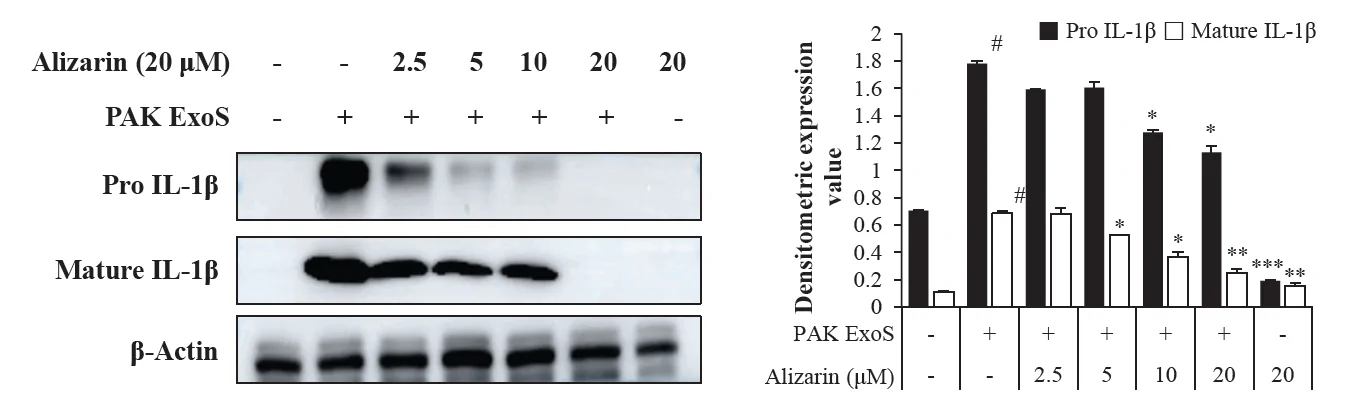
Levels of conversion to mature IL-1β from pro-IL-1β by alizarin. Western blotting was performed to detect the two types of IL-1β formation. After infection for 1 h with PAK ExoS, H292 cells were washed with DPBS containing tobramycin and supplied RPMI 1640 (0.1% FBS) for 1 h. (A) Each group exhibited decreases in pro IL-1β and mature IL-1β, which is converted by caspase-1 when alizarin is added. Each band was quantified using ImageJ and is represented by a number indicating the difference between the control and nontreated bands. PAK-ΔSTmt-pUCP18 (PAK ExoS; PAK-ΔSTmt recovered the ExoS gene).

We investigated IL-1β to confirm these results since IL-1β is initially produced and converted to a mature form by caspase-1 and secreted. It was observed that IL-1β in the pro-form decreased according to the concentration of alizarin; it was also confirmed that IL-1β in the mature form decreased (Fig. 6). These findings demonstrate that alizarin is effective in cell infection caused by *P. aeruginosa* and argue that the effect is a result of regulating the inflammatory response induced by LPS and caspase-1 activated by ExoS.

## Discussion

*P. aeruginosa* causes many diseases by infecting hosts, but only antibiotics can be used to cure it because no effective drugs have been identified. Currently, pneumonia caused by *P. aeruginosa* highly contributes to mortality among the many diseases induced by *P. aeruginosa* infection. The T3SS is one of several secretion systems of Gram-negative bacteria and has a unique characteristic of delivering effector proteins directly inside the host. Secreted ExoS effector protein can attack the host actin cytoskeleton and cause cell death, which can interrupt immune cells (such as macrophages and neutrophils). Thus, infection can increasingly develop until the host is overwhelmed (Cornelis and Wolf-Watz, 1997; Hauser 2009).

Alizarin is known as a therapeutic for various diseases in China. However, the relationship between alizarin and *P. aeruginosa* has not been reported, so we observed pathogenetic factors, including a growth curve. Biofilms contribute to avoiding the attack of immune cells by forming on mucosal surfaces; it has been reported that the T3SS translocon helps to form a biofilm when bacteria settle at the epithelial barrier (Dongari-Bagtzoglou, 2008; Du Toit, 2015; Moser et al., 2021; Tran et al., 2014). We confirmed decreased ExoS-Flag and biofilm formation when *P. aeruginosa* was incubated with alizarin (Fig. 1C, 1D, 1E, and 1F). We assume that additional T3SS factors, such as translocon proteins, will be affected by alizarin, assuming an unknown causal relationship between alizarin, ExoS and biofilm due to the decreases in ExoS and biofilm. To prove this result, we performed real-time PCR at the bacterial level after the incubation of alizarin with PAK ExoS (Fig. 2). Interesti ngly, *popD*, a translocon gene that forms cell membrane pores (Fig. 2B), was significantly inhibited. What stood out was the level of *ExsA*, which regulates the entire T3SS gene (Fig. 2A) (Armentrout and Rietsch, 2016; Tang et al., 2018).

We hypothesize that this expression profile is due to the inhibition of *popD* following the reduction of *ExsA* by alizarin. This is because the expression of *pscF*, which encodes the same T3SS needle complex, was also decreased (Fig. 2C) (Burkinshaw and Strynadka, 2014). Since alizarin inhibited ExoS by ecreasing *ExsA* at the bacterial level, we expected that it could inhibit ExoS by the same mechanism in infected H292 cells. The current well-known model of infection by *P. aeruginosa* in the lung is pneumonia (Hauser, 2009; Wagener et al., 2020a, 2020b). Its infection strategy is very lethal and difficult to counteract for the host. *P. aeruginosa*, which is capable of movement via flagella, readily contacts lung epithelial cells and stimulates TLR4 via LPS, resulting in the activation of NF-κB (Mori et al., 1999; Park et al., 2022; Preciado et al., 2005). Activated NF-κB produces and secretes proinflammatory cytokines (IL-1β, IL-6, IL-18), leading to the excessive recruitment of immune cells (such as macrophages and neutrophils) (Al Moussawi and Kazmierczak, 2014; Sun et al., 2020). At this time, *P. aeruginosa* directly contacts immune cells to neutralize the host's immune response and injects ExoS to destroy the actin skeleton, thereby causing cell death (Henriksson et al., 2002; Pederson et al., 1999; Sun and Barbieri, 2004). In this situation, the host lacks effective defenses against *P. aeruginosa*, and the initial inflammation leads to pneumonia. To determine whether alizarin can inhibit ExoS and the inflammatory response of *P. aeruginosa* at the H292 cell level, we evaluated the cytotoxicity of alizarin and the survival of the cells by alizarin in infected H292 cells (Fig. 3A and 3B). The results confirmed that alizarin did not affect the toxicity or cell viability at any concentration. However, ExoS levels and proinflammatory cytokines in infected H292 cells were significantly decreased according to the alizarin concentration (2.5, 5, 10, and 20 μM) (Fig. 3C, 4A, 4B, and 4C). These results suggested that alizarin may not only be involved in *P. aeruginosa* T3SS but may also affect signaling pathways that induce inflammation. Therefore, we infected H292 cells treated with alizarin with PAK ExoS and observed the protein levels of NF-κB factors (Fig. 5). Alizarin treatment decreased the levels of p-p65. To understand whether alizarin precisely inhibits p65 phosphorylation, we observed the level of p65 translocation into the nucleus and found that alizarin treatment resulted in a significant inhibitory effect at Fig. 5B. Therefore, the reduced translocation of p65 may indicate that the production of proinflammatory cytokines is reduced. In addition, it could be expected that the IL-1β produced could not be converted to a mature form due to the reduction of caspase-1 by alizarin. To substantiate this hypothesis, we confirmed the pro- and mature forms of IL-1β, and alizarin showed inhibitory effects at both levels (Fig. 6).

**Fig. 7.**
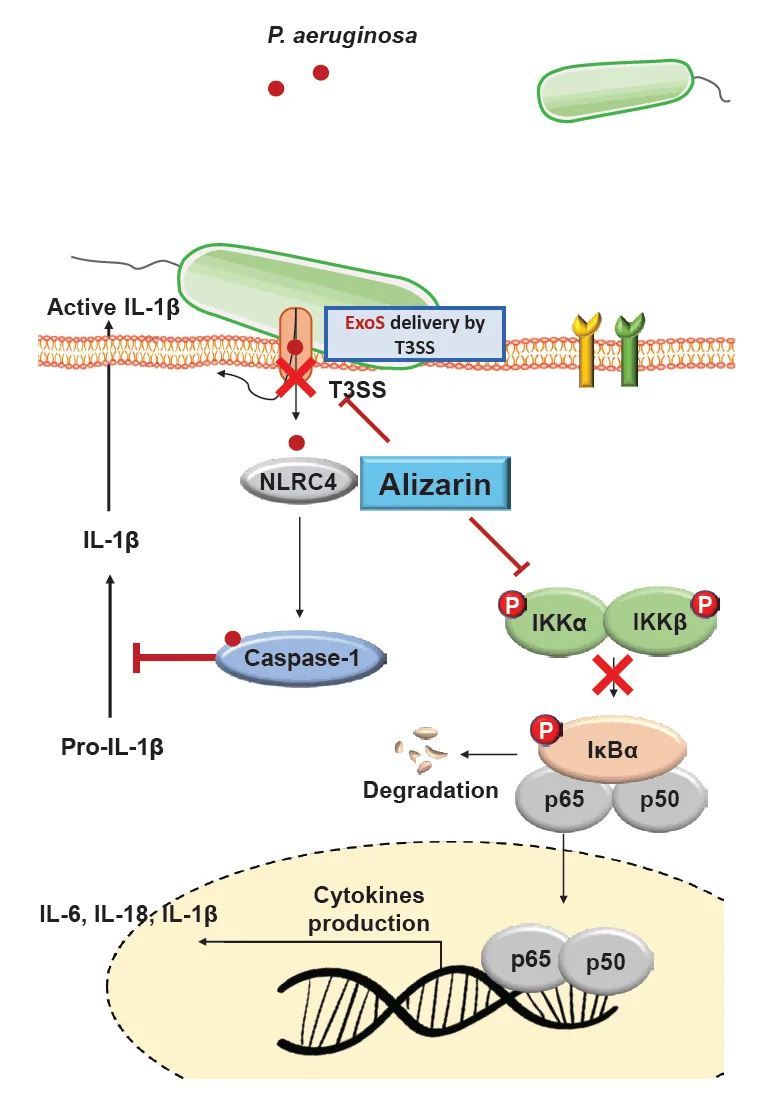
Schematic diagram illustrating the mechanisms underlying the anti-inflammatory effects of alizarin. *P. aeruginosa*, *Pseudomonas aeruginosa*; T3SS, Type three secretion system; ExoS, Exoenzyme S; NF-κB, nuclear factor-κB; IKKα, NF-κB activation involves IκB kinase alpha; IKKβ, NF-κB activation involves IκB kinase beta; IκB-α, inhibitor of NF-κB; NLRC4, NLR family CARD domain-containing protein 4; IL-1β, interleukin-1beta; IL-6, interleukin-6; IL-18, interleukin-18

These results demonstrate that alizarin not only exhibits the same T3SS inhibitory effect in cells as it did at the bacterial level but can also attenuate the inflammatory response (Fig. 7). Unfortunately, we have not been able to elucidate whether alizarin precisely interacts with T3SS and inhibits the inflammatory response at the cellular level. We plan to conduct further experiments to evaluate these possibilities. Based on the results thus far, we argue that alizarin may have a significant effect on current *P. aeruginosa* lung infections.

## Conclusion

In our experiments, alizarin reduced the level of T3SS used by *P. aeruginosa* by regulating the mRNA expression of ExsA. In addition, pro-inflammatory cytokines increased by NF-κB production in infected H292 cells were inhibited upon the addition of alizarin. This effect was shown by reducing the level of ExoS delivered to the host by alizarin and inhibiting the activity of the induced caspase-1. Collectively, this study showed that alizarin treatment attenuated infection and inflammation during *P. aeruginosa* infection in H292 cells through the interruption of bacteria from interacting with H292 cells via ExoS delivery (Fig. 7). Therefore, we propose that alizarin is an attractive therapeutic for *P. aeruginosa* infection in the lung.

## Figures and Tables

**Table 1.** List of all strains and plasmids used in this study

Strain or plasmid	T3SS discription	Antibiotics	Source or reference
Strain			
PAK	Wild type, functional T3SS		University of Florida
PAK exsA::Ω (p34)	PAK with the malfunction of T3SS transcription control gene loci with Ω cassette	Sp^200^, Sm^200^, Cb^150^	From Dr. Ha (Korea University)
PAK-ΔSTmt	Double effector mutant: functional needle and translocon apparatus without known effector	Cb^150^	From Dr. Ha (Korea University)
PAK-ΔSTmt-pUCP18 (PAK ExoS)	A Functional Effector ExoS In PAK-ΔSTmt	Cb^150^	
PAK exoST::Ω/pHW0225 (p137)	Double mutated ExoS-FLAG	Sp^200^, Cb^150^, Gm^150^	From Dr. Ha (Korea University)
Plasmid			
PAK-ΔSTmt-pUCP18	Cloning vector (Vector only 4,557 bp)	Cb^150^	From JWP (Natural Medicine Research Center)
PAK-ΔSTmt-pUCP18PAKexoS [ExoS]	pUCP18 PAKexoS		From JWP (Natural Medicine Research Center)
PAK-ΔSTmt-pUCP18PAKexoT [ExoT]	pUCP18 PAKexoT		From JWP (Natural Medicine Research Center)

**Table 2.** Sequences of the reverse transtription PCR primers used in the current study

Bacteria primer		
	Forward	Reverse
ExoS	CAG GCT GAA CAG GTA GTG AAG	TTC AGG GAG GTG GAG AGA TAG
ExsA	AAG GAG CCA AAT CTC TTG	CTT GTT TAC CCT GTA TTC G
popD	ATC CAG TCC TTC GTC CAG AT	TCC TCG ACC TTC TGC TTC T
pscF	TCA ACG ACG CGA TCA AGG	CCG TCG AGT TGA TGT TGT AGA T
16S	CAA AAC TAC TGA GCT AGA GTA CG	GCC ACT GGT GTT CCT TCC TA
Human primer		
IL-1β	GGG CCT CAA GGA AAA GAA TC	TTC TGC TTG AGA GGT GCT GA
IL-18	TGC ATC AAC TTT GTG GCA AT	ATA GAG GCC GAT TTC CTT GG
IL-6	GAC AGC CAC TCA CCT CTT CA	AGT GCC TCT TTG CTG CTT TC
gapDH	CCC TCC AAA ATC AAG TGG	CCA TCC ACA GTC TTC TGG
IL-8	ACT GAG AGT GAT TGA GAG TGG AC	AAC CCT CTG CAC CCA GTT TTC
